# Donor DNA Utilization During Gene Targeting with Zinc-Finger Nucleases

**DOI:** 10.1534/g3.112.005439

**Published:** 2013-04-01

**Authors:** Kelly J. Beumer, Jonathan K. Trautman, Kusumika Mukherjee, Dana Carroll

**Affiliations:** Department of Biochemistry, University of Utah School of Medicine, Salt Lake City, Utah 84112-5650

**Keywords:** Gene targeting, zinc-finger nucleases, conversion tracts, nonhomologous end joining, homologous recombination

## Abstract

Gene targeting is the term commonly applied to experimental gene replacement by homologous recombination (HR). This process is substantially stimulated by a double-strand break (DSB) in the genomic target. Zinc-finger nucleases (ZFNs) are targetable cleavage reagents that provide an effective means of introducing such a break in conjunction with delivery of a homologous donor DNA. In this study we explored several parameters of donor DNA structure during ZFN-mediated gene targeting in *Drosophila melanogaster* embryos, as follows. 1) We confirmed that HR outcomes are enhanced relative to the alternative nonhomologous end joining (NHEJ) repair pathway in flies lacking DNA ligase IV. 2) The minimum amount of homology needed to support efficient HR in fly embryos is between 200 and 500 bp. 3) Conversion tracts are very broad in this system: donor sequences more than 3 kb from the ZFN-induced break are found in the HR products at approximately 50% of the frequency of a marker at the break. 4) Deletions carried by the donor DNA are readily incorporated at the target. 5) While linear double-stranded DNAs are not effective as donors, single-stranded oligonucleotides are. These observations should enable better experimental design for gene targeting in *Drosophila* and help guide similar efforts in other systems.

Zinc-finger nucleases (ZFNs) (Carroll 2011), their close relatives the transcription activator-like effector nucleases (TALENs) (Bogdanove and Voytas 2011), and engineered homing endonucleases (Stoddard 2011) are proving very effective at enhancing the efficiency of targeted gene modifications. They all work by making double-strand breaks (DSBs) in genomic DNA at sites determined by their designed DNA recognition domains. In the cases of ZFNs and TALENs, recognition is mediated by modules that can be assembled in arbitrary combinations to bind specifically to a wide range of DNA sequences. Because the nuclease domain appended to these binding modules must dimerize (Bitinaite *et al.* 1998; Smith *et al.* 2000), pairs of ZFNs or TALENs are required to achieve cleavage.

DSBs made by these nucleases are repaired by cellular mechanisms, most prominently homologous recombination (HR) and nonhomologous end joining (NHEJ) (Figure 1). When the goal is targeted gene replacement by HR, a manipulated donor DNA is provided, and the efficiency of its utilization determines the success of the experiment. We showed earlier that disabling the major NHEJ pathway in *Drosophila* greatly improves the recovery of HR products (Beumer *et al.* 2008; Bozas *et al.* 2009). In this study, we examine several other parameters of donor utilization, including homology requirements, conversion tracts, inclusion of large deletions, and the usefulness of short single-stranded DNA donors. The results show how cellular activities in this particular experimental context affect gene targeting outcomes and should provide guidance for analogous studies in other systems.

**Figure 1:**
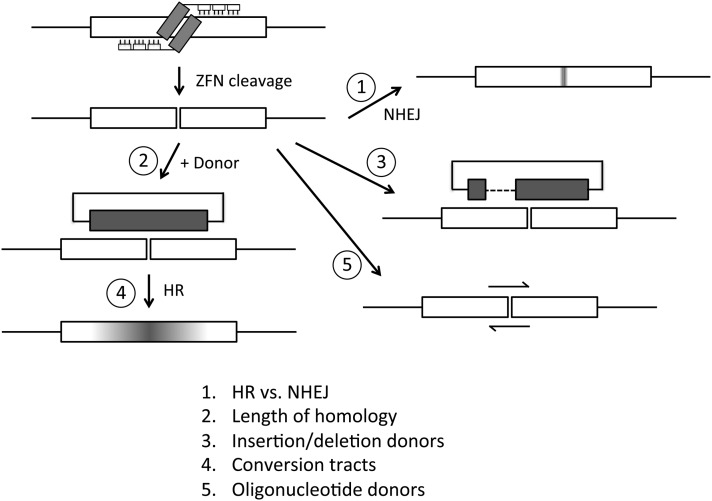
Illustration of ZFN cleavage and various repair possibilities. The types of donor DNAs used are shown, and the issues addressed in this study are numbered and listed at the bottom.

## Materials and Methods

### Stocks

Several different stocks were used as recipients for embryo injections. Canton-S and a stock carrying the third chromosome from the *w^1118^* stock were used as wild types. Two *lig4* stocks carried the *lig4^169^* mutation, either in a wild-type or *w^1118^* background (Bozas *et al.* 2009; McVey *et al.* 2004b), and some injections were performed in a stock carrying the *lig4^EP385^* mutation (Bloomington Stock Center). Stocks and crosses to reveal new *rosy* (*ry*) mutations were as described previously (Beumer *et al.* 2006). Stocks for inducing ZFN mutagenesis by heat shock were as described previously (Beumer *et al.* 2006).

### Plasmids and donor construction

The construction of plasmids to serve as templates for *in vitro* synthesis of *ry* ZFN RNAs, pCS2-ryA and pCS2-ryB, has been previously described (Beumer *et al.* 2008), as has the construction of the 4.2-kb donor (Beumer *et al.* 2006) and the 7.5-kb donor [7.46-kb symmetrical donor in Beumer *et al.* (2008)]. The shorter donors were constructed using these plasmids as a starting point as follows.

The 0.2-kb, 0.5-kb, and 1.0-kb donors were constructed by amplifying appropriately sized fragments from the 4.2-kb donor, using LA *Taq* polymerase (TaKaRa, Clontech, Mountain View, CA) and cloning the resulting fragments into pGEM-T (Promega, Madison, WI). The fragments were amplified using the following oligonucleotides (see Table S1 for sequences): 0.2 kb, ry-*Pvu*I-F and ry-9350-R; 0.5 kb, ry-8981-F and ry-9532-R; and 1.0 kb, ry-8788-F and ry-9720-R.

The 2.1-kb donor was constructed by digesting the 4.2-kb donor with *Eco*RI and *Nde*I (removing the 5′-most 2.1 kb), blunting, and re-ligating the shortened vector.

Deletion donors were constructed using the 7.5-kb donor as a starting point. To construct the AA donor, the 7.5-kb donor plasmid was digested with *Age*I and *Afl*II, blunted with DNA polymerase I Klenow fragment (New England Biolabs, Ipswich, MA), and re-ligated. This created a 347-bp deletion starting 269 bp downstream of the target site. The BX donor was constructed by digesting the 7.5-kb donor with *Xba*I and *Bsp*DI, then blunting and religating as above, creating a 1232-bp deletion beginning at the target site. The SA donor was constructed by digesting the 7.5-kb donor with *Afl*II and *Sgr*AI and blunting and religating as above, creating a 1307-bp deletion beginning 699 bp upstream and ending 608 bp downstream of the target site. The BP donor was constructed by digesting the 7.5-kb donor with *Bss*HII and *Pml*I, then blunting and religating as above, creating a 1089-bp deletion ending 825 bp upstream from the target site. All donors were verified by sequencing at the University of Utah DNA sequencing Core Facility.

Oligonucleotides were synthesized at the University of Utah DNA/Peptide Core Facility, except for those used for TaqMan analysis, which were made by Integrated DNA Technologies (Coralville, IA).

### Injection and mutagenesis

RNA injections were performed as previously described (Beumer *et al.* 2008). Briefly, RNAs were transcribed *in vitro* from plasmids carrying the ryA and ryB ZFN coding sequences. The RNAs were mixed at 350 µg/ml each and injected into syncytial embryos by standard procedures. Each separate injection set involved approximately 200 embryos. Both plasmid and oligonucleotide donors were injected at a concentration of 500 µg/ml in the same solution as the ZFN RNAs. Some experiments with oligonucleotide donors were performed with TALENs directed to the *ry* target, rather than ZFNs (K. J. Beumer, unpublished data). Experiments involving heat shock induction of ZFNs were carried out as previously described (Beumer *et al.* 2006).

The total number of offspring scored in each experiment was approximately 80 per injected parent in the Canton-S stock. With the *lig4* stocks, the total number of offspring was approximately 50 per parent; and because of the segregation of the *w^−^* allele in many cases, only the female offspring of female *lig4* parents could be scored for *ry* mutations.

Caution should be exercised in interpreting the detailed quantitation in the experiments. While the broad conclusions have been verified by multiple iterations of each experimental regimen, there is some variability in quantitative detail among repeats. Despite efforts to standardize conditions, the factors responsible have not been identified but could include the health of the injected embryos, the quality of the injection needle, and/or the properties of the RNA/DNA solutions being injected.

### Molecular analysis

Mutations at the *ry* locus were recovered and analyzed as described previously (Beumer *et al.* 2006), with the following modifications. HR mutants were tested for the AA deletion by amplification with the primers ry-*Pvu*I-F and ry-genomic-R (Table S1). If the deletion is present, the resulting fragment is 787 bp, while the corresponding wild-type fragment is 1130 bp. HR mutants were tested for the BX deletion by amplification with ry-*Pvu*I-F and ry-10320-R. The wild-type fragment was 1396 bp; the deletion was 169 bp. To test for the BP deletion, a polymerase chain reaction (PCR) assay was performed with ry-506-7640-F and ry-*Pvu*I-R in mutants that had already tested positive for HR. A wild-type fragment was 2230 bp; the mutant fragment was 1143 bp. SA deletions were identified by amplification with ry-*Nde*I-F and ry-genomic-R; the wild-type fragment was 2117 bp; the HR fragment was 810 bp.

### Conversion tracts

Initial conversion tract experiments were performed in a stock that carried a third chromosome derived from the *w^1118^* stock used for transformations of the heat shock-driven ZFNs. Five sites were chosen where a polymorphism changed a restriction site between the donor and the target chromosomes (see Table S1 for PCR primers). For each site, the region of the polymorphism was amplified and the resulting fragment digested with the appropriate restriction enzyme. The digested DNA was then visualized on an agarose gel and examined for either the donor or the target digestion pattern.

Some experiments with the 7.5-kb donor were performed in a *lig4^169^* stock that lacked the *Bst*NI polymorphism. An additional polymorphism was identified at position +3341 relative to the ZFN cut site between the donor and the *ry* locus in both the Canton-S and the *lig4^169^* stocks. In addition to restriction enzyme digestion, some loci were tested with alternative assays. The *Pvu*II site at position −2812, the *Kpn*I site at −460, the *Dpn*II site at +391, and the site at +3341 were assayed using a customized TaqMan single-nucleotide polymorphism endpoint genotyping assay (Applied Biosystems) using an ABI 7900HT real-time detection instrument and analyzed with Applied Biosystems SDS version 2.3 software at the University of Utah Genomic Core. Primers for these assays were designed by the University of Utah Core facility. The *Hae*III site at position +842 also introduces a 2-bp deletion in the target. PCR primers ry-842-FLP-F and ry-842-FLP-R (Table S1) were used to amplify fragments of 155 bp from the target and 157 bp from the donor. These were run on an Applied Biosystems 3130 × l capillary electrophoresis instrument (36 cm capillary, POP 7 polymer) and analyzed with Applied Biosystems GeneMapper version 3.7 software. An additional polymorphism was identified between the donor and the *lig4^169^ ry* locus at position +1985. This polymorphism was not present in the wild-type stock. The polymorphism introduces a 7-bp deletion in the target in comparison to the donor. The PCR primers ry-1985-FLP-F and ry-1985-FLP-R were used to amplify a 160-bp fragment from the target sequence and a 167-bp fragment from the donor. They were analyzed as described above.

## Results

### Experimental design

All experiments were performed in *Drosophila melanogaster*. With one exception, ZFN mRNAs and donor DNAs were delivered by direct injection into embryos (Beumer *et al.* 2008). We showed earlier that linear double-stranded DNAs were ineffective as donors in this protocol, so donors were injected as parts of circular plasmids, with the exception of single- and double-stranded oligonucleotides, which were injected as linear molecules. Two types of host strains were used for the injections: one was wild type for DNA repair and one lacked DNA ligase IV, a key component of the major NHEJ pathway. Earlier experiments showed that HR was significantly enhanced in the latter background (Beumer *et al.* 2008; Bozas *et al.* 2009).

The target in all cases was a sequence in the third exon of the *rosy* (*ry*) gene, and the corresponding ZFN pair was that described previously (Beumer *et al.* 2006; Beumer *et al.* 2008). All donor DNAs carried sequence changes that created a null mutation in the target (Beumer *et al.* 2006; Beumer *et al.* 2008). In addition, the donors were altered so that they could not be cleaved by the ZFNs, either when free or following their incorporation into the genomic locus.

Injected flies were raised to adults and then crossed with partners carrying a deletion (*ry^506^*) covering most of the *ry* gene. New *ry* mutants among the progeny were identified by eye color. This procedure revealed only germline mutations and scored each parental *ry* allele individually. Thus, the proportion of offspring that carried new *ry* mutations in each experiment was equivalent to the frequency of mutation at the target. Many of the mutants were subjected to molecular analysis to distinguish HR products from those created by NHEJ. The designation % HR is the proportion of all mutants analyzed that scored as HR products.

### Homology requirements

Based on earlier studies (Kurkulos *et al.* 1994; McVey *et al.* 2004a; Nassif *et al.* 1994), including our characterization of ZFN-induced mutagenesis in various repair mutant strains (Bozas *et al.* 2009), we assumed that most HR products arose by a pathway called synthesis-dependent strand annealing (SDSA) (Symington 2002). This mechanism is illustrated in Supporting Information, Figure S1. It involves resection of 5′ strand ends at both sides of the break, invasion of homologous sequences in the donor, copying donor sequences by extension from the invading 3′ end, withdrawal of the extended strand, annealing with the other end at the break, and resolution by some combination of polymerase, nuclease, and ligase activities.

Studies of homologous recombination in many organisms have shown that mechanisms depending on strand invasion require a minimum length of homology between donor and recipient. In eukaryotes, this lower limit is typically in the range of 200 to 400 bp (Waldman 2008) and is presumably due to a need for adequate stability of intermediate structures involving a displaced strand (see Figure S1). We explored this limit in ZFN-induced HR in *Drosophila* by testing donors of various lengths incorporated into a circular plasmid with a backbone of approximately 3 kb (Figure S2). The homology in all cases, except that of the 4.2-kb donor, was approximately equally distributed on the two sides of the ZFN-induced break.

**Figure 2:**
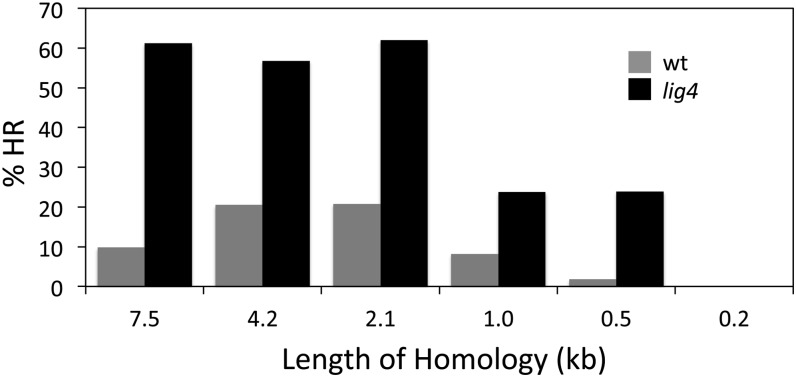
Influence of the length of homology between donor and target DNAs on the proportion of all new mutants that are the result of homologous recombination (%HR). Gray bars are data obtained for the wild-type (*lig4^+^*) host, and black bars are for the *lig4* mutant host. Additional data are provided in Table 1.

As shown in Figure 2 and Table 1, HR products were recovered with all donor lengths from 7.5 to 0.5 kb but not with the 0.2-kb donor. As seen previously, the proportion of HR mutants was quite low in the wild-type background: 20% or less for long donor homologies and dropping off for total homologies of 1.0 kb and, particularly, 0.5 kb. This parameter was substantially higher in the *lig4* mutant, in the vicinity of 60% HR for homologies of 2.1 kb or longer. The % HR dropped to 24% for 1.0-kb and 0.5-kb donors and was undetectable with only 0.2 kb of homology.

**Table 1:** Effect of donor homology length

Donor Length	Host	Parents	Yielders	Mutants	No. of Mutants/Parent	Tested	HR	% HR
0.2 kb	CS	84	18	246	2.9	110	0	0
	*lig4*	109	9	86	0.8	34	0	0
0.5 kb	CS	108	27	415	3.8	166	3	2
	*lig4*	167	34	278	1.7	155	37	24
1.0 kb	CS	73	22	409	5.6	122	10	8
	*lig4*	111	23	258	2.3	105	25	24
2.1 kb	CS	120	47	736	6.1	240	50	21
	*lig4*	155	26	189	1.2	108	67	62
4.2 kb	CS	489	275	6745	13.8	1231	252	20
	*lig4*	462	152	3688	7.9	998	567	57
7.5 kb	CS	296	151	4111	13.9	852	84	10
	*lig4*	221	68	1542	7.0	369	226	61

Donors of the indicated lengths were tested in wild-type Canton S (CS) flies or a *lig4* mutant. The total number of parents tested, number of those yielding mutants, and total number of mutants recovered are listed, as is the average number of mutants per parent. The number of individual mutants tested for NHEJ *vs.* HR is shown, then the number of those that incorporated the donor sequence (HR). Finally the percentage of those tested that were products of HR is listed (HR/tested × 100).

Some variability was observed in the proportion of injected flies that yielded new mutants, and this feature and the total yield of mutants were always lower in *lig4* mutants than in the wild type (Table 1). We conclude that the lower limit of homology that will support efficient HR from an injected donor in this system is between 200 and 500 bp, in good agreement with the results obtained with other eukaryotic systems (Waldman 2008). In addition, there is a modest step down in efficiency between 2 kb and 1 kb of homology.

### Conversion tracts

The mutation carried in the donor DNAs in the preceding experiments was located precisely at the site of the break in the target. An important issue to address is how far from the cut site are donor sequences efficiently incorporated at the target? What is typically observed in break-induced HR is that donor incorporation decreases with increasing distance from the site of the break. This is observed, for example, in meiotic recombination in fungi, a process known to be initiated by DSBs (Paques and Haber 1999). In the case of gene targeting with ZFNs, once a pair of nucleases has been established for a given genomic target, it would be useful to be able to introduce sequence changes at various sites within that locus without the need to develop new cleavage reagents specific for each site.

**Figure 3:**
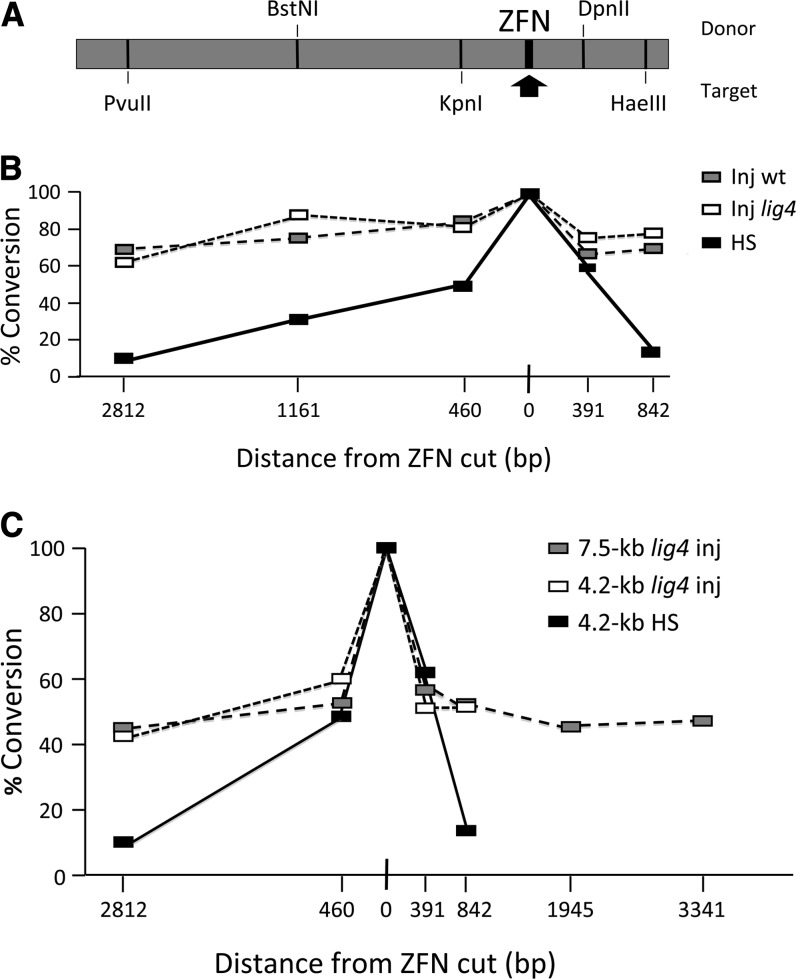
Conversion tracts during homologous recombination. (A)The 4.2-kb donor showing polymorphic sites between donor and target that define restriction enzyme sites. The ZFN site is indicated by an arrow. (B) %Conversion is the proportion of HR products that acquired sequences from the 4.2-kb donor at each site. The ZFN cut site is set to 100% as all HR events must capture donor sequence there. Black boxes are results from experiments in which ZFN expression and donor excision were induced in larvae with a heat shock (HS). Open and gray boxes are results from experiments with injected embryos, either wild type (Inj wt) or *lig4* mutants (Inj *lig4*). (C) As in panel B, but showing independent experiments with 4.2-kb and 7.5-kb donors. HS data from panel B are repeated for comparison.

With this goal in mind, we examined conversion tracts during ZFN-induced targeting at the *ry* locus. This was done first using the more elaborate protocol we initially used in *Drosophila* (Beumer *et al.* 2006; Bibikova *et al.* 2003). ZFN coding sequences were established as transgenes driven by a heat shock promoter. The donor DNA was also integrated in the genome, but was excised as a linear molecule following a brief heat shock. Donor and target DNAs, both derived from natural wild-type *ry* alleles, differed in sequence at a number of sites, some of which created restriction site polymorphisms between the two (Figure 3A). When HR products were recovered, the relevant regions were amplified by PCR and tested for sensitivity to the indicated restriction enzymes.

As shown in Figure 3B (HS data), the frequency of gene conversion decreased as a function of distance on both sides of the ZFN-induced break. Conversion was low but still detectable, approximately 10% of maximum, nearly 3 kb from the break. In fact, this plot is virtually superimposable on data generated previously for conversion during repair of a break induced by *P* element excision (Gloor *et al.* 1991; Nassif and Engels 1993).

We repeated this type of experiment using the embryo injection protocol and circular donor DNAs. Both 4.2-kb and 7.5-kb donors were used. In this case, the conversion tracts were much broader, both in wild-type and *lig4* mutant backgrounds (Figure 3, B and C). Conversion was approximately 50% at sites 3 kb away on either side of the ZFN-induced break. The difference from the results of the HS experiment shown in Figure 3B is presumably due to the stage of germline development, the method of delivery of the ZFNs and donor, and/or linear *vs.* circular donor configurations.

Analysis of the conversion tracts individually showed that most were continuous, although a few showed interspersed donor and target markers (Figure S3, A–C). The latter represent only 3 of 68, 4 of 66, and 3 of 78 tracts in the three experiments and may be due to short-track mismatch correction in heteroduplexes formed during homologous recombination.

**Figure 4:**
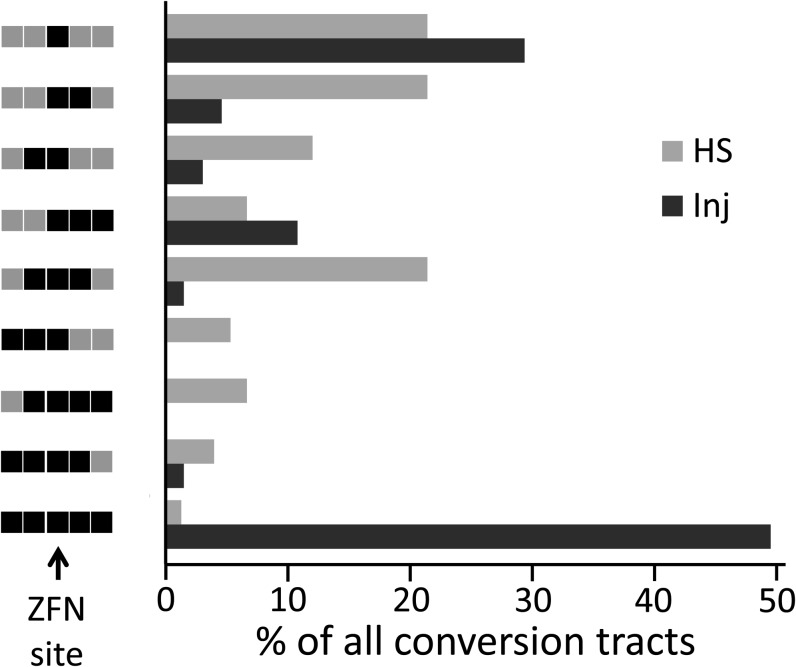
Histogram of data for the 4.2-kb donor in injection (Inj) and HS experiments shown in Figure 3C. The 5 sites assayed in both cases are illustrated on the left as having donor (black) or target (gray) sequence(s); the ZFN site (arrow) is in the middle and is from donors in all cases, as only HR products were included. The proportions of all tracts represented by each type are indicated by the lengths of the bars in the histogram.

In the HS experiment, most conversion tracts were short, and the frequency of long tracts was quite low (Figure 4). In contrast, tract lengths showed a bimodal distribution in the injection experiment. Nearly half the tracts showed donor sequence over the entire assayed length, while the remainder were very short, with conversion only at the cut site or nearby. This is shown for the 4.2-kb donor in Figure 4, and it is obvious for the injected 7.5-kb donor as well (Figure S3B).

One other feature differed between the heat shock and embryo injection data. In the injections, a larger proportion of the HR products showed two-sided conversion tracts, while from in the HS experiment, more were one-sided or simply involved the ZFN cut site (Figure S3). These differences probably reflect the same phenomenon noted in the preceding paragraph, *i.e.*, more of the tracts in the injection experiments showed complete conversion to donor sequence.

The yield of mutants and the proportion due to HR are quite comparable between the HS and injection protocols (Beumer *et al.* 2008). Injections are much simpler, and the conversion tracts are longer, so this would normally be the method of choice.

### Introducing deletions

**Figure 5:**
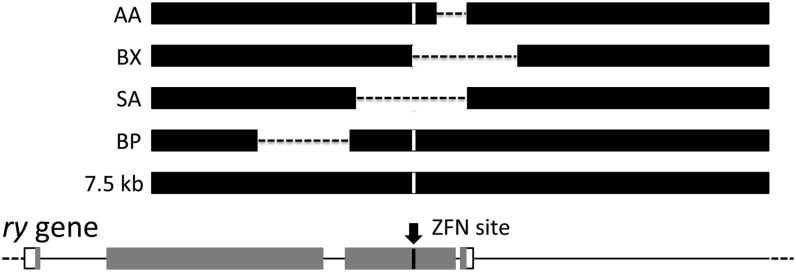
Deletion donors. The genomic *ry* locus is shown, with exons as rectangles and introns and intergenic sequences as lines. Protein coding sequences are gray. The ZFN target sequence is shown by an arrow. Donor homologies are shown as black rectangles, dashes indicate sequences deleted, and the substitution at the ZFN site is indicated by a vertical white line. Data for the deletion donors are provided in Table 2.

In addition to testing small polymorphisms, we tested the incorporation at the target of larger sequence alterations carried by the donor. We constructed several different deletions, as shown in Figure 5, all in the context of the 7.5-kb donor. The AA deletion removed 347 bp starting 269 bp from the ZFN site; the BP deletion removed 1089 bp separated from the ZFN site by 825 bp. The deletion in BX started at the ZFN site and extended for 1232 bp on one side. Deletions in donors AA, BX, and BP were recovered with reasonable efficiencies (Table 2).

**Table 2:** Data for deletion donors

Donor	Host	Parents	Yielders	Mutants	No. of Mutants/Parent	Tested	HR	% HR	% w/Δ
7.5 kb	CS	52	17	214	4.1	88	3	3	NA
	*lig4*	52	13	103	2.0	69	14	20	NA
AA	CS	204	47	618	3.0	362	26	7	0
	*lig4*	171	45	605	3.5	256	47	18	26
BP	CS	109	38	675	6.2	266	14	5	36
	*lig4*	53	23	359	6.8	137	66	48	30
BX	CS	60	21	237	4.0	153	18	12	100
	*lig4*	95	25	257	2.7	117	51	44	100
SA	CS	80	9	133	1.7	94	0	0	NA
	*lig4*	69	15	160	2.3	89	0	0	NA

Data for experiments with donors carrying deletions (see Figure 5). Entries are as in Table 1. The final column shows the percentage of all homologous recombinants that carried the deletion present in the donor. This figure is necessarily 100 for the BX donor, which has homology immediately adjacent to the ZFN cut only on one side. NA, not applicable.

No homologous recombinants were recovered with the SA donor, either in wild-type or *lig4^−^* backgrounds. This deletion extends for approximately 700 bp and 600 bp on each side of the ZFN site. The failure to utilize this donor as a template for repair may reflect a need for homology near one of the ends at the break to initiate SDSA.

### Oligonucleotide donors

**Figure 6:**
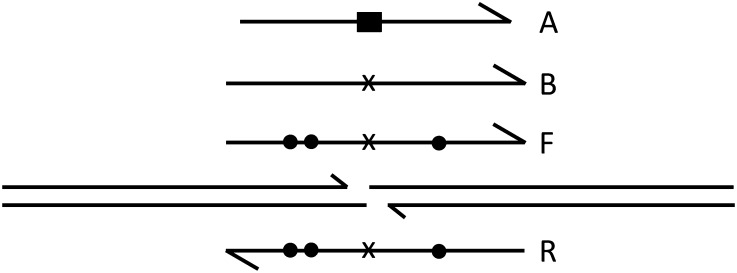
Single-stranded oligonucleotide donors. The ZFN-cut target is illustrated with longer lines. Half arrowheads indicate 3′ ends. Null mutations in the oligonucleotides are shown as a black rectangle (deletion-substitution) or an x (single-base deletion). Dots indicate single-base substitutions. Oligonucleotides A, B, and F have the same polarity; oligonucleotide R has reverse polarity. Data for oligonucleotide donors are provided in Table 3.

While double-stranded donors with extensive homology to the target serve well and offer the prospect of introducing changes over substantial distances, synthetic oligonucleotides are simpler to prepare and have been shown to be efficiently incorporated in mammalian cells (Chen *et al.* 2011; Radecke *et al.* 2010) and in zebrafish embryos (Bedell *et al.* 2012). We tested whether such molecules could also serve as donors in the *Drosophila* embryo injection protocol. To do so, we made several oligonucleotide donors (Figure 6 and Figure S4) with the following characteristics: donor A consisted of 101 nt corresponding to the sense strand of the longer donors, carrying the substitution that replaces the zinc finger recognition sites with two in-frame stop codons and an *Xba*I site (Beumer *et al.* 2006); a B donor consisted of 111 nt, corresponding to the same strand of the donor but with a simple 1-nt deletion in the spacer between ZF binding sites; this null mutation is very rare among ZFN-induced NHEJ mutations and, by shortening the spacer to 5 bp, sharply reduces the efficiency of ZFN cutting (our unpublished results); an F donor, consisting of 111 nt, same as B, but with single-nucleotide changes on either side of the ZFN cut; an R donor containing 111 nt, the complement of the F donor; an F+R donor, consisting of 111 bp, an annealed pair of the oligonucleotides F and R.

All of the single-stranded oligonucleotides were successfully used as donors, and the frequencies were higher in the *lig4* mutant background (Table 3). The absolute frequencies obtained were somewhat variable and might have depend on the concentration of injected DNA, a parameter that we did not test extensively.

**Table 3:** Data for oligonucleotide donors

Oligonucleotide	Host	Parents	Yielders	Mutants	No. of Mutants/Parent	Tested	HR	% HR
A	CS	80	34	541	6.8	178	2	1
	*lig4*	22	8	51	2.5	38	4	11
B	CS	not done						
	*lig4*	57	11	147	2.6	75	26	35
F	CS	154	27	291	1.9	115	22	19
	*lig4*	89	12	119	1.3	57	17	30
R	CS	143	25	513	3.6	107	17	8
	*lig4*	136	51	833	6.1	329	52	16
F+R	CS	102	10	120	1.2	69	6	9
	*lig4*	65	16	87	1.3	52	3	6

The oligonucleotides are those shown in Figure 6 and Figure S4. F+R is the annealed duplex of the single strands F and R. Entries are as in Table 1.

Contrary to our experience with longer double-stranded DNAs (Beumer *et al.* 2008), even the annealed oligonucleotide pair F+R yielded some apparent HR products. However, we cannot rule out the possibility that some single strands remained after annealing, and these products were recovered from only a single parent in each of two independent experiments. Thus, the earlier conclusion (Beumer *et al.* 2008) that linear dsDNA is ineffective as a donor may stand.

The F and R oligonucleotides were marked on either side of the ZFN-induced break (Figure 6) in the hope that the HR products would reveal information about the mechanism of donor copying. The results, however, showed no significant difference in marker recovery between the two configurations (Figure S5). In addition, the multiple mismatches between donors F and R and the target did not seem to affect their utility (Table 3).

## Discussion

In this study we examined in detail several parameters that affect donor utilization in ZFN-stimulated gene targeting experiments. Our principal findings were: 1) the minimum amount of homology necessary to support efficient gene replacement is between 200 and 500 bp with a circular donor DNA molecule; 2) conversion tracts are quite broad in events occurring in injected *Drosophila* embryos, reaching several kb on either side of the ZFN-induced break; 3) large deletions in the donor DNA are readily incorporated at the target; and 4) single-stranded oligonucleotides serve as effective donors.

Each of these conclusions is important for designing donor DNAs for future gene targeting applications in *Drosophila*. While the particular lessons derived are specific for this specific experimental system, the considerations are important for all nuclease-mediated targeting experiments. Because all events after target cleavage reflect activities available in cells for double-strand break repair, different systems will have different properties with respect to these parameters. In principle, gene targeting outcomes can be modified by manipulating relevant cellular activities, as we have done by using *lig4* mutants to increase HR relative to NHEJ.

For *Drosophila*, the frequency of NHEJ mutagenesis, the enhancement of HR in a *lig4* background, and the utility of both plasmid and oligonucleotide donors reported here encourage the use of ZFNs for modifying essentially any genomic locus. The ryA:ryB ZFN pair is the most active of the ones we have tested, but we have achieved very good levels of targeted mutagenesis at other loci by using designed ZFNs (Beumer *et al.* 2008) and TALENs (K. J. Beumer, unpublished data). In many cases, new mutants were identified by molecular screening, when no visible phenotype was available.

### Homology requirements

The minimum length of matched sequence required for efficient homologous recombination has sometimes been called minimum effective processing segment (MEPS) (Shen and Huang 1986). It is usually cited as being 50–100 bp in bacteria and 200–400 bp in eukaryotes (Waldman 2008), and it is thought to correspond to the minimum amount of homology necessary to stabilize an intermediate structure(s) during HR. Our observations are in line with those obtained in other eukaryotes (*i.e.*, a circular donor must have more than 200 bp of homology with the target to support HR with reasonable efficiency in conjunction with a ZFN-induced break). In the embryo injection system, homology lengths greater than 1 kb provide additional efficiency, particularly in a *lig4* mutant background.

As has been demonstrated in mammalian cells (Chen *et al.* 2011; Radecke *et al.* 2010) and in zebrafish embryos (Bedell *et al.* 2012), single-stranded oligonucleotides of approximately 100 bases also serve effectively as donors. As with longer donors, frequencies were higher in the absence of DNA ligase IV. It is difficult to make comparisons in efficiency with double-stranded circular donors as the oligonucleotides were injected at much higher molarity. Nonetheless, with our experimental conditions, oligonucleotides served as donors for HR as effectively as the longer DNAs. The oligonucleotides are easier to generate for local mutagenesis but obviously cannot convert sequences over a long distance.

### Conversion tracts

Our observations regarding conversion tracts have particular importance. When ZFN expression and donor excision were induced by heat shock in *Drosophila* larvae, the incorporation of donor sequences at the target declined with distance from the break, falling to approximately 10% of maximum at 3 kb away. This result corresponds well to earlier determinations of conversion tracts during homologous repair following *P* element excision (Gloor *et al.* 1991; Nassif and Engels 1993). When ZFN mRNAs and a donor DNA were injected into fly embryos, however, the conversion tracts were much longer. Donor sequences more than 3 kb from the break were incorporated at approximately 50% of maximum.

The broader tracts recovered from embryo events presumably reflect differences in available DNA repair activities and possibly differences in HR mechanism. Available evidence indicates that most homologous DSB repair in the larval germline proceeds by SDSA (Figure S1) (Bozas *et al.* 2009; Kurkulos *et al.* 1994; McVey *et al.* 2004a; Nassif *et al.* 1994). This particular issue has not been addressed directly in syncytial embryos. The long tracts observed are consistent with either long heteroduplex intermediates that form during HR repair or extensive degradation of target sequences at the break. If the former, and if SDSA is still the primary pathway, we can envision one or more of the following contributions: 1) synthesis tracts might be much longer in embryonic germline due to more processive DNA polymerization; 2) in embryos, the capacity for strand withdrawal is somehow limited; and 3) mismatch repair is less active in embryos. The last of these possibilities seems less likely, as the absence of efficient mismatch repair in all cases studied leads to postrecombination segregation of markers but not to extension of conversion tracts. It is also possible that an alternative pathway for repair dominates in embryos. For example, single-strand annealing (SSA), which is a minor pathway in larvae (Bozas *et al.* 2009), can produce very long heteroduplex intermediates (Lehman *et al.* 1994).

Whatever the reason, long conversion tracts are very desirable during gene targeting. Once a ZFN pair, or another targeted nuclease, has been validated for a target within a locus, it can be used to introduce subtle or dramatic sequence changes from a suitably designed donor over long distances, without the need to develop additional cleavage reagents. Whenever conversion tracts have been examined in cultured mammalian cells, they have proven to be relatively short, on the order of a few hundred base pairs (Elliott *et al.* 1998). Discovering what limits these tracts in such cells or what allows long ones in *Drosophila* embryos should enable appropriate manipulations that expand the tracts usefully in many organisms.

### Other donor configurations

Consistent with our analysis of conversion tracts based on very localized polymorphisms, we also found that deletions of hundreds of base pairs were readily converted from donors into the *ry* target. This was true for deletions that retained homology close to the ZFN-induced DSB but not for one that removed matching sequences for several hundred base pairs on either side of the break. We presume that the invading end from the break must be able to locate homology in the donor within some minimum distance. Homology to one end is sufficient, as the BX donor was effectively used. Short mismatches at the break ends do not interfere, as all donors (except SA) carried a deletion of 23 bp and an insertion of 12 bp relative to the target sequence.

With the oligonucleotide donors, the efficiency of HR was somewhat lower for the version carrying the deletion/insertion opposite the cut site than for the one with a single base pair deleted (Table 1). This observation is based on small numbers, however, and thus needs additional confirmation.

We also tried one donor carrying a large insertion, of a mini-white gene. While this donor was used successfully (data not shown), expression of the inserted gene was apparently suppressed due to natural regulation of *ry* expression (Reaume *et al.* 1989), hampering our ability to generate quantitative data. Insertions at other loci presumably would not be subject to this effect.

## Supplementary Material

Supporting Information
