# Supplementary material for: Donor DNA Utilization During Gene Targeting with Zinc-Finger Nucleases
Source: G3 (Bethesda). 2013 Apr 1;3(4):657–64. doi: 10.1534/g3.112.005439 (PMC3618352; doi:10.1534/g3.112.005439)
Supplement: Supporting Information [file supp_3_4_657__index.html]

Donor DNA Utilization during Gene Targeting with Zinc-finger Nucleases — Donor DNA Utilization During Gene Targeting with Zinc-Finger Nucleases — Supporting Information 

# Donor DNA Utilization During Gene Targeting with Zinc-Finger Nucleases

## Supporting Information for Beumer *et al.*, 2013

**Files in this Data Supplement:**

- Supporting Information - Figures S1-S5 and Table S1 (PDF, 383 KB)
- Figure S1 - Illustration of the synthesis-dependent strand annealing (SDSA) mechanism of homologous recombination (PDF, 87 KB)
- Figure S2 - Illustration of the donors used to determine homology length requirements (PDF, 129 KB)
- Figure S3 - Diagrams of individual conversion tracts from the experiments summarized in Figures 3C and 4 (PDF, 176 KB)
- Figure S4 - Sequences of oligonucleotides used as donors (PDF, 81 KB)
- Figure S5 - Histogram showing conversion of polymorphisms from forward (F) and reverse (R) oligonucleotide donors into the target (PDF, 96 KB)
- Table S1 - Oligonucleotides used in this study (PDF, 58 KB)
