# Supplementary material for: Donor DNA Utilization During Gene Targeting with Zinc-Finger Nucleases
Source: G3 (Bethesda). 2013 Apr 1;3(4):657–64. doi: 10.1534/g3.112.005439 (PMC3618352; doi:10.1534/g3.112.005439)
Supplement: Supporting Information [file supp_g3.112.005439_005439SI.pdf]

G3 · Genes | Genomes | Genetics

Donor DNA Utilization during Gene Targeting with Zinc-finger Nucleases

Kelly J. Beumer, Jonathan K. Trautman, Kusumika Mukherjee and Dana Carroll

Department of Biochemistry

University of Utah School of Medicine

Salt Lake City, UT, USA

DOI 10.1534/g3.112.005439

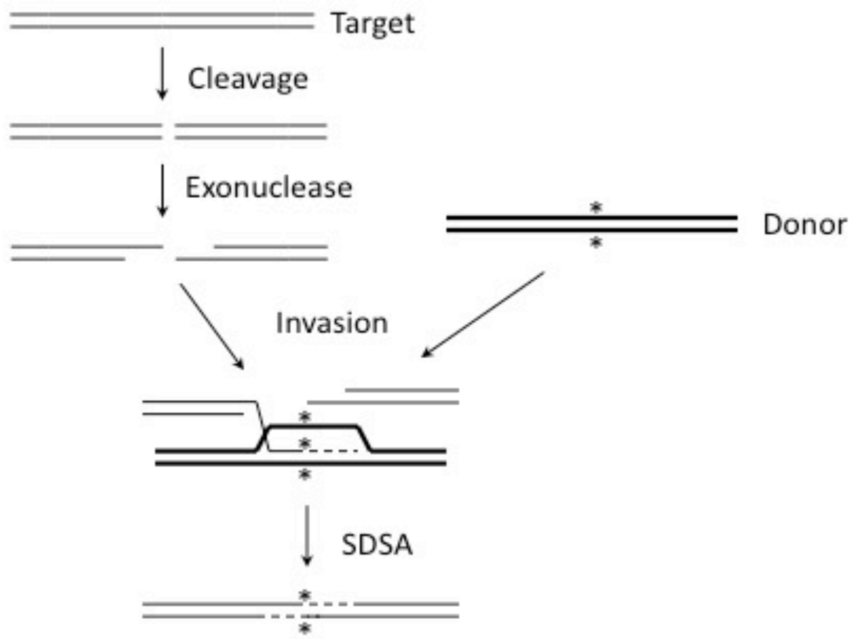

Figure S1 Illustration of the synthesis-dependent strand annealing (SDSA) mechanism of homologous recombination. Following a double-strand break, 5' ends are resected by exonuclease action. One of the resulting 3'-ending, single-stranded tails invades homologous sequence in the donor and is extended by DNA polymerase. The invading end withdraws and pairs with the other end from the break, and the junction is completed by polymerase, nuclease and ligase activities. Any mutations (*) copied from the donor are incorporated at the target by this process.

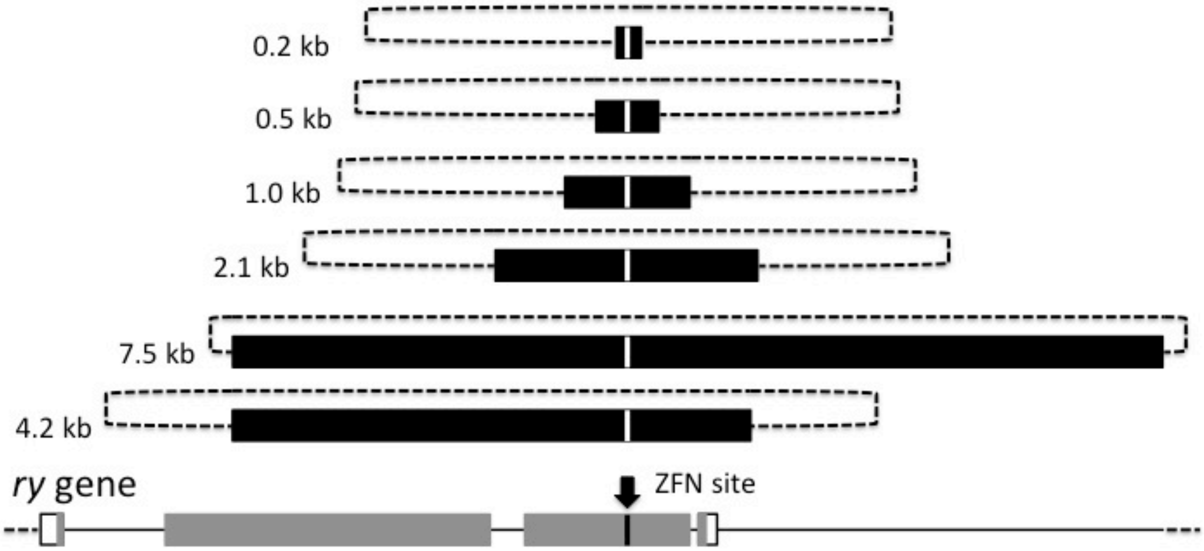

Figure S2 Illustration of the donors used to determine homology length requirements. The wild type *ry* gene is shown at the bottom, with exons as rectangles and coding sequences shaded gray and the ZFN recognition site in black. The donor homologies are shown as black rectangles, with the mutated ZFN site in white. The plasmid vector backbones are shown as dotted lines.

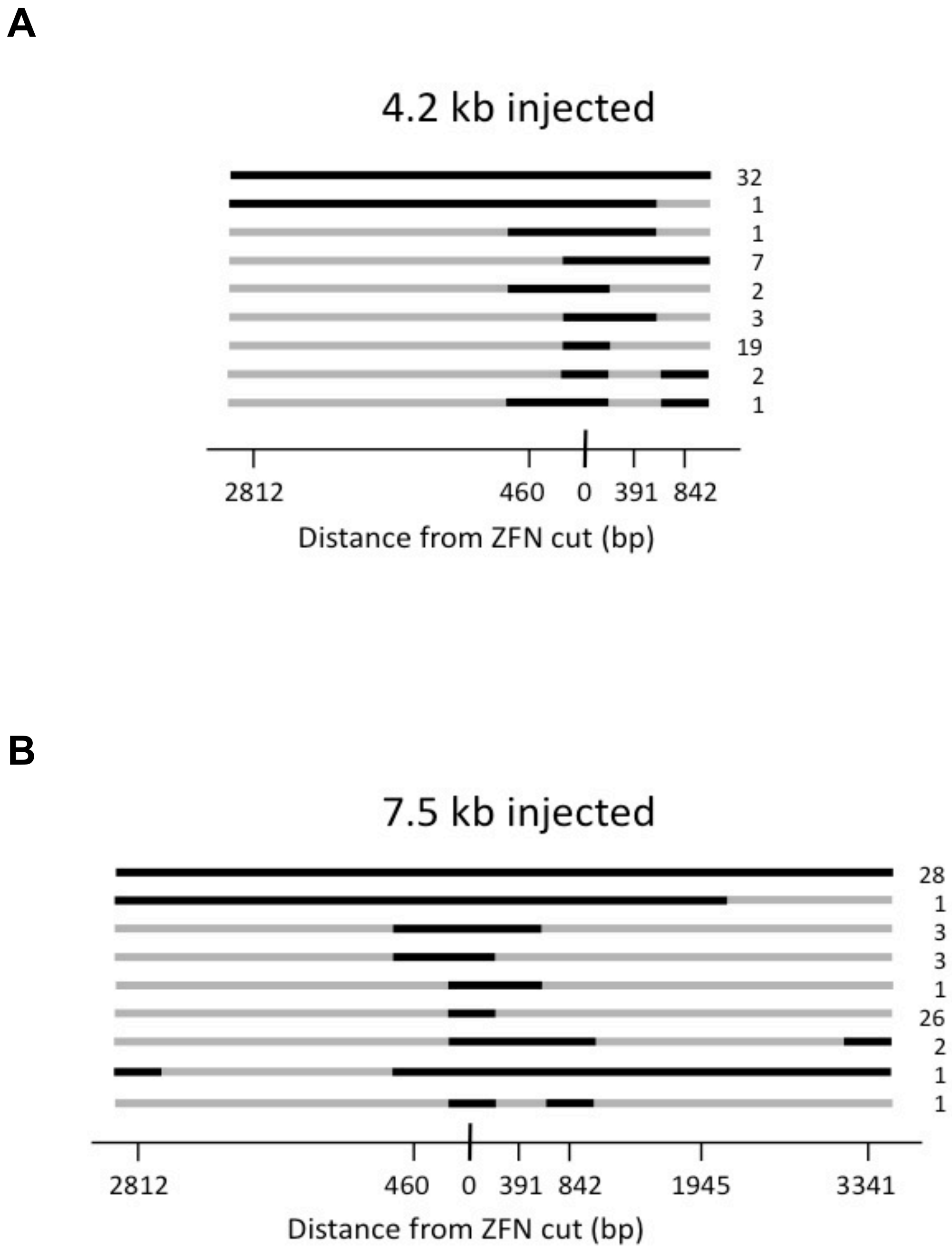

A
4.2 kb injected
32
1
1
7
2
3
19
2
1
2812
460
0
391
842
Distance from ZFN cut (bp)
B
7.5 kb injected
28
1
3
3
1
26
2
1
1
2812
460
0
391
842
1945
3341
Distance from ZFN cut (bp)

C

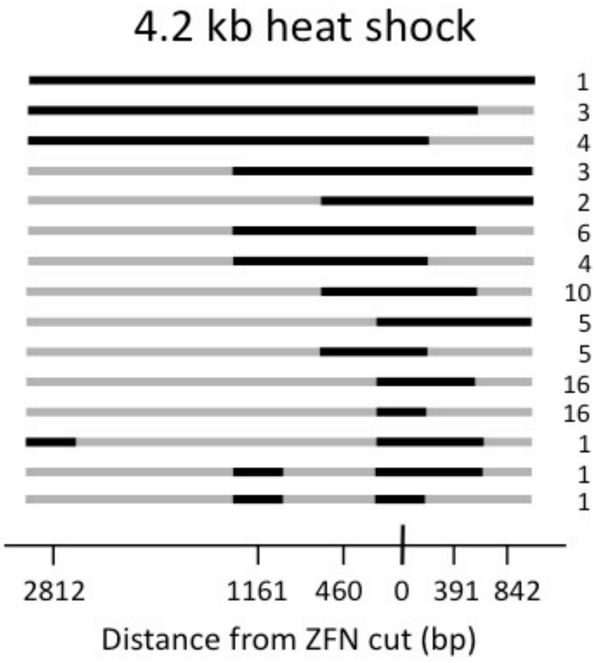

Figure S3 Diagrams of individual conversion tracts from the experiments summarized in Figures 3C and 4. **A**, tracts from injection of the 4.2-kb donor. **B**, tracts from injection of the 7.5-kb donor. **C**, tracts from the HS experiment with the 4.2-kb donor. In each panel the sites assayed and their distance from the ZFN cut are indicated at the bottom. Black bars show the extent of donor sequence found in the HR products; gray bars show remaining target sequence. The number of instances is given at the right of each tract type.

WT sequence
TTGGATATCACCCGGAAACGAATCCCAATGCTCGCACCTAT**AGCTACTACACGAATGGC**
GTGGGAGTCACTGTGGTAGAGATCGATTGCCTGACTGGCGACCATCAGGTGCT

Oligo A
TTGGATATCACCCGGAAACGAATCCCAATGCTCGCACCTAT**TGATAATCTAGAA**GTCAC
TGTGGTAGAGATCGATTGCCTGACTGGCGACCATCAGGTGCT

Oligo B
TTGGATATCACCCGGAAACGAATCCCAATGCCCGCACCTAT**AGCTACTACAC_AATGGC**
GTGGGAGTCACTGTGGTAGAGATCGATTGCCTGACTGGCGACCATCAGGTGCT

Oligo F *Eco*RI
TTGGATATCACCCGGAAAC**GAATTC**CAATGCCCGCACCTAT**AGCTACTACAC_AATGGC**
GTGGGAGTCACTGTGGTAG**CGATCG**ATTGCCTGACTGGCGACCATCAGGTGCT
*Pvu*I

Oligo R
AGCACCTGATGGTCGCCAGTCAGGCAAT**CGATCG**CTACCACAGTGAC**TCCCACGCCATT**
_GTGTAGTAGCTATAGGTGCGGGCATTG**GAATTC**GTTTCCGGGTGATATCCAA

Figure S4 Sequences of oligonucleotides used as donors. Each sequence starts at the 5' end. The ZFN target is highlighted in bold blue type; the sequence that replaces it in Oligo A is in bold green type. The underlined space in Oligos B, F, and R shows the site of a single nucleotide deletion. The substitutions in Oligos F and R are shown in red, and the restriction enzyme recognition sites created by two of them (identified for Oligo F) are in bold type. One additional polymorphism in B, F and R is shown in red and underlined. Oligo R is the complement of Oligo F.

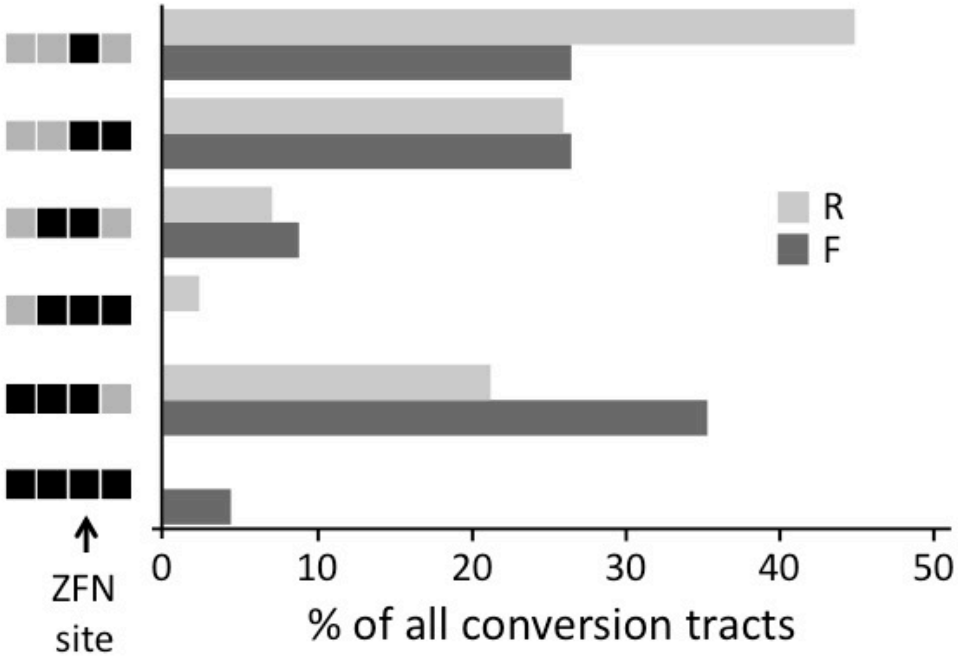

Figure S5 Histogram showing conversion of polymorphisms from forward (F) and reverse (R) oligonucleotide donors into the target. As in Figure 4, the boxes at the left represent the 4 sites at which the donors differ from the target. A black box indicates donor sequence in the HR product; a gray box indicates target sequence. The donor mutation within the ZFN site is indicated with an arrow. The patterns are very similar, with a preponderance of simple ZFN-site and one-sided conversions. In particular, there is no significant bias with either donor for conversions on the left or right of the ZFN cut. The number of independent conversion tracts scored was 23 for the F oligo and 43 for the R oligo.

Table S1 Oligonucleotides used in this study (see also Figure S5).

Oligo name	DNA sequence (5'->3')	Used in
ry-PvuI-F	CAAGGCGTATTTCGATCGGGTCAGCC	0.2-kb donor construction<br>AA, BX deletion detection
ry-9350-R	AATAGCCGGATTCAGGCTAGAGC	0.2-kb donor construction
ry-8981-F	CGGAAACGGCCACGGATAAAGTA	0.5-kb donor construction
ry-9532-R	GTAGGCTGACATTGAACTCCCCG	0.5-kb donor construction
ry-8788-F	AATCGGGAGAATCGCTGGCGGAAAC	1.0-kb donor construction
ry-9720-R	GCAATTCGAATGCGTGCCGATGTGG	1.0-kb donor construction<br>*Kpn*I site polymorphism
ry-genomic-R	GCGAAGATCTCGTCTCATAACCTGAG	AA deletion detection<br>SA deletion detection<br>*Hae*III site polymorphism<br>*Dpn*II site polymorphism
ry-10320-R	ACACACTTACCAATCAGCGGACG	BX deletion detection
ry-506-7640-F	CACCAGCTCGTGGAGCGCGTG	BP deletion detection
ry-PvuI-R	GGCTGACCCGATCGAAATACGCCTTG	BP deletion detection
ry-NdeI-F	GGAAAATCCATATGTAGGTTTCTTTATGCATACC	SA deletion detection
ry-9085-F	GGAATGGCCGTACTGGATGCGTG	*Hae*III site polymorphism<br>*Dpn*II site polymorphism
ry-EcoRI-F	GAGCGCAGCGAATTCCAGCCCTTG	*Pvu*II site polymorphism
ry-1460-R	CCGAGCGGCTCTTTGGGCC	*Pvu*II site polymorphism
ry-BstNI-F	CACCAAGCTGGATGCCAGTGAAG	*Bst*NI site polymorphism
ry-NdeI-R	GGTATGCATAAAGAAACCTACATATGGATTTTCC	*Bst*NI site polymorphism
ry-6000-F	GTGGGCTTCACCAAGGAGGGTC	*Kpn*I site polymorphism
ry-842-FLP-F	5HEX/CCTTTTAGATTAAGAAAAATTCAATGG	*Hae*III site polymorphism
ry-842-FLP-R	TTCATCGGAAAACTTGCTCA	*Hae*III site polymorphism
ry-1985-FLP-F	56-FAM/CCATAACGGCAGCACACTG	Polymorphism at +195
ry-1985-FLP-R	CTCAACGCCACCAATCAA	Polymorphism at +195

The last 4 entries were used in assays by high-resolution length analysis of the PCR products, and the forward primer (F) carried a fluorescent label in each case.
