# Supplementary material for: Donor DNA Utilization During Gene Targeting with Zinc-Finger Nucleases
Source: G3 (Bethesda). 2013 Apr 1;3(4):657–64. doi: 10.1534/g3.112.005439 (PMC3618352; doi:10.1534/g3.112.005439)
Supplement: Supporting Information [file supp_g3.112.005439_FigureS1.pdf]

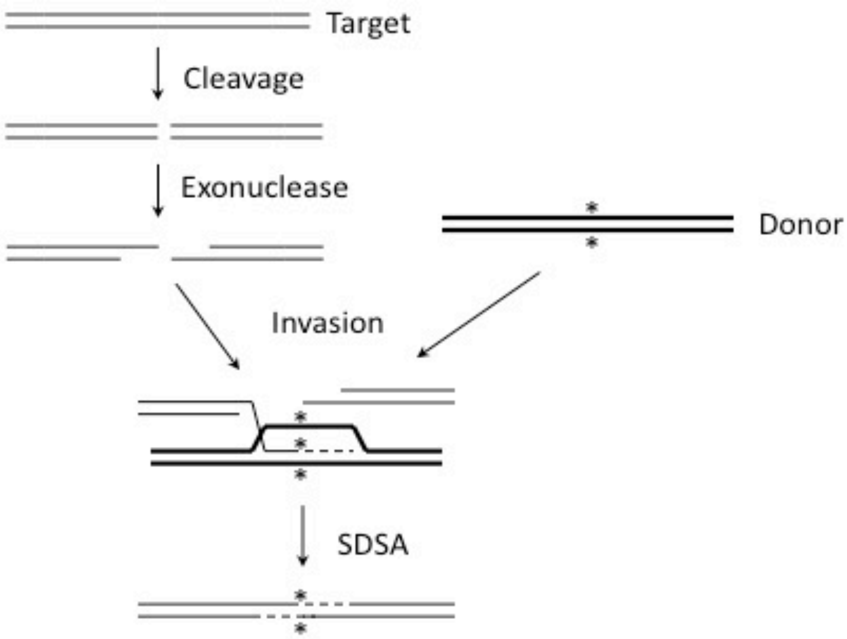

Figure S1 Illustration of the synthesis-dependent strand annealing (SDSA) mechanism of homologous recombination. Following a double-strand break, 5' ends are resected by exonuclease action. One of the resulting 3'-ending, single-stranded tails invades homologous sequence in the donor and is extended by DNA polymerase. The invading end withdraws and pairs with the other end from the break, and the junction is completed by polymerase, nuclease and ligase activities. Any mutations (*) copied from the donor are incorporated at the target by this process.
