# Supplementary material for: Donor DNA Utilization During Gene Targeting with Zinc-Finger Nucleases
Source: G3 (Bethesda). 2013 Apr 1;3(4):657–64. doi: 10.1534/g3.112.005439 (PMC3618352; doi:10.1534/g3.112.005439)
Supplement: Supporting Information [file supp_g3.112.005439_FigureS2.pdf]

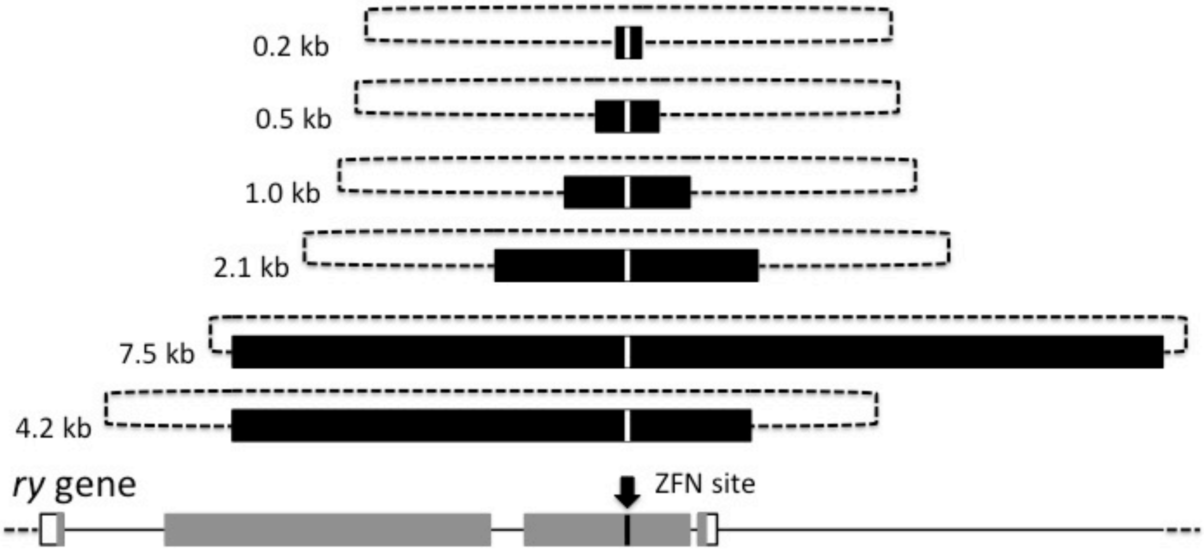

Figure S2 Illustration of the donors used to determine homology length requirements. The wild type *ry* gene is shown at the bottom, with exons as rectangles and coding sequences shaded gray and the ZFN recognition site in black. The donor homologies are shown as black rectangles, with the mutated ZFN site in white. The plasmid vector backbones are shown as dotted lines.
