# Supplementary material for: Donor DNA Utilization During Gene Targeting with Zinc-Finger Nucleases
Source: G3 (Bethesda). 2013 Apr 1;3(4):657–64. doi: 10.1534/g3.112.005439 (PMC3618352; doi:10.1534/g3.112.005439)
Supplement: Supporting Information [file supp_g3.112.005439_FigureS3.pdf]

A

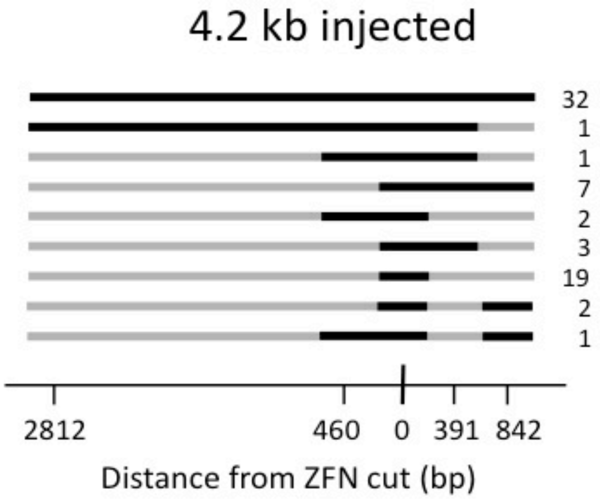
4.2 kb injected
32
1
1
7
2
3
19
2
1
2812
460
0
391
842
Distance from ZFN cut (bp)

B

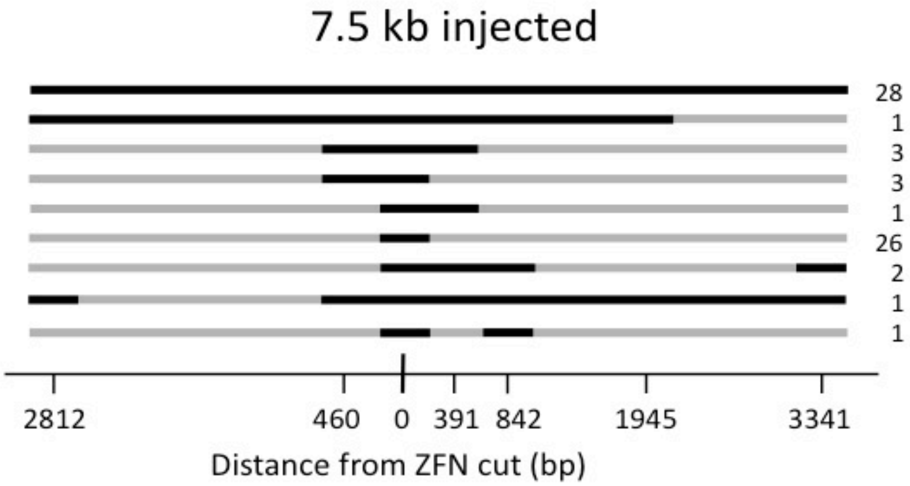
7.5 kb injected
28
1
3
3
1
26
2
1
1
2812
460
0
391
842
1945
3341
Distance from ZFN cut (bp)

C

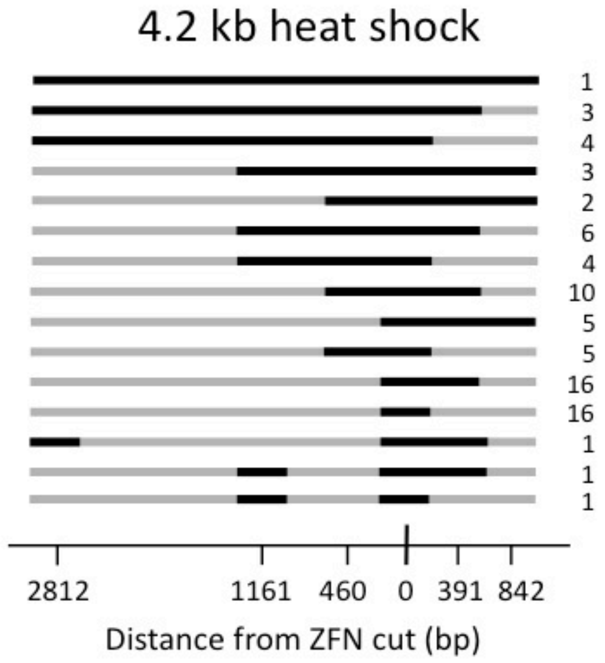

Figure S3 Diagrams of individual conversion tracts from the experiments summarized in Figures 3C and 4. **A**, tracts from injection of the 4.2-kb donor. **B**, tracts from injection of the 7.5-kb donor. **C**, tracts from the HS experiment with the 4.2-kb donor. In each panel the sites assayed and their distance from the ZFN cut are indicated at the bottom. Black bars show the extent of donor sequence found in the HR products; gray bars show remaining target sequence. The number of instances is given at the right of each tract type.
