# Supplementary material for: Donor DNA Utilization During Gene Targeting with Zinc-Finger Nucleases
Source: G3 (Bethesda). 2013 Apr 1;3(4):657–64. doi: 10.1534/g3.112.005439 (PMC3618352; doi:10.1534/g3.112.005439)
Supplement: Supporting Information [file supp_g3.112.005439_FigureS4.pdf]

WT sequence
TTGGATATCACCCGGAAACGAATCCCAATGCTCGCACCTAT**AGCTACTACACGAATGGC**
**GTGGGA**GTCACTGTGGTAGAGATCGATTGCCTGACTGGCGACCATCAGGTGCT

Oligo A
TTGGATATCACCCGGAAACGAATCCCAATGCTCGCACCTAT**TGATAATCTAGAA**GTCAC
TGTGGTAGAGATCGATTGCCTGACTGGCGACCATCAGGTGCT

Oligo B
TTGGATATCACCCGGAAACGAATCCCAATGCCCGCACCTAT**AGCTACTACAC_AATGGC**
**GTGGGA**GTCACTGTGGTAGAGATCGATTGCCTGACTGGCGACCATCAGGTGCT

Oligo F *Eco*RI
TTGGATATCACCCGGAAAC**GAATTC**CAATGCCCGCACCTAT**AGCTACTACAC_AATGGC**
**GTGGGA**GTCACTGTGGTAG**CGATCG**ATTGCCTGACTGGCGACCATCAGGTGCT
*Pvu*I

Oligo R
AGCACCTGATGGTCGCCAGTCAGGCAAT**CGATCG**CTACCACAGTGAC**TCCCACGCCATT**
**_GTGTAGTAGCT**ATAGGTGCGGGCATTG**GAATTC**GTTTCCGGGTGATATCCAA

**Figure S4** Sequences of oligonucleotides used as donors. Each sequence starts at the 5’ end. The ZFN target is highlighted in bold blue type; the sequence that replaces it in Oligo A is in bold green type. The underlined space in Oligos B, F, and R shows the site of a single nucleotide deletion. The substitutions in Oligos F and R are shown in red, and the restriction enzyme recognition sites created by two of them (identified for Oligo F) are in bold type. One additional polymorphism in B, F and R is shown in red and underlined. Oligo R is the complement of Oligo F.
