# Supplementary material for: Donor DNA Utilization During Gene Targeting with Zinc-Finger Nucleases
Source: G3 (Bethesda). 2013 Apr 1;3(4):657–64. doi: 10.1534/g3.112.005439 (PMC3618352; doi:10.1534/g3.112.005439)
Supplement: Supporting Information [file supp_g3.112.005439_FigureS5.pdf]

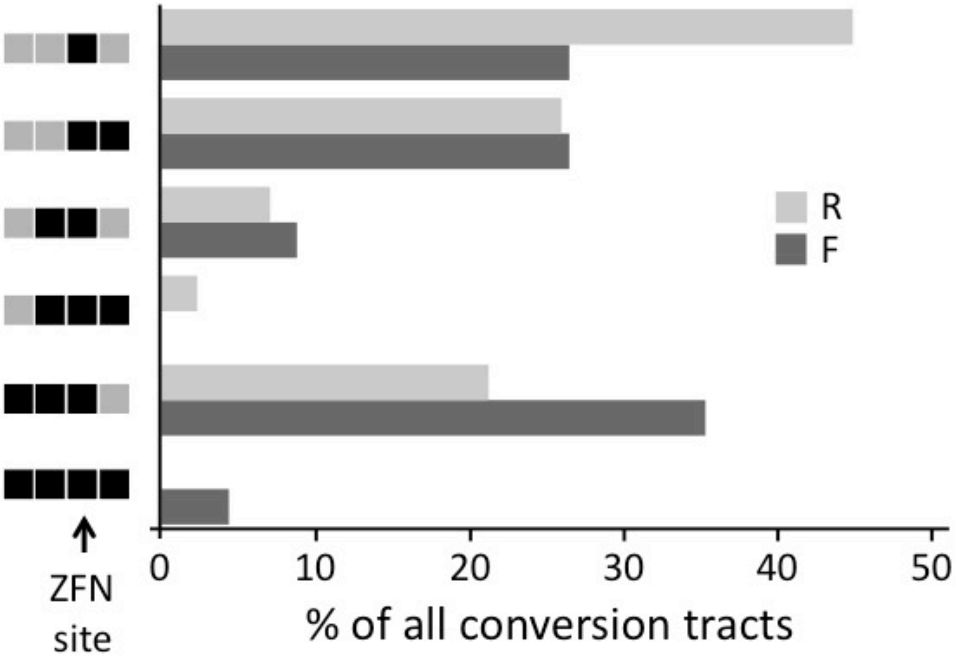

Figure S5 Histogram showing conversion of polymorphisms from forward (F) and reverse (R) oligonucleotide donors into the target. As in Figure 4, the boxes at the left represent the 4 sites at which the donors differ from the target. A black box indicates donor sequence in the HR product; a gray box indicates target sequence. The donor mutation within the ZFN site is indicated with an arrow. The patterns are very similar, with a preponderance of simple ZFN-site and one-sided conversions. In particular, there is no significant bias with either donor for conversions on the left or right of the ZFN cut. The number of independent conversion tracts scored was 23 for the F oligo and 43 for the R oligo.
