# Supplementary material for: Donor DNA Utilization During Gene Targeting with Zinc-Finger Nucleases
Source: G3 (Bethesda). 2013 Apr 1;3(4):657–64. doi: 10.1534/g3.112.005439 (PMC3618352; doi:10.1534/g3.112.005439)
Supplement: Supporting Information [file supp_g3.112.005439_TableS1.pdf]

Table S1 Oligonucleotides used in this study (see also Figure S5).

Oligo name	DNA sequence (5'->3')	Used in
ry-PvuI-F	CAAGGCGTATTTCGATCGGGTCAGCC	0.2-kb donor construction<br>AA, BX deletion detection
ry-9350-R	AATAGCCGGATTCAGGCTAGAGC	0.2-kb donor construction
ry-8981-F	CGGAAACGGCCACGGATAAAGTA	0.5-kb donor construction
ry-9532-R	GTAGGCTGACATTGAACTCCCCG	0.5-kb donor construction
ry-8788-F	AATCGGGAGAATCGCTGGCGGAAAC	1.0-kb donor construction
ry-9720-R	GCAATTCGAATGCGTGCCGATGTGG	1.0-kb donor construction<br>*Kpn*I site polymorphism
ry-genomic-R	GCGAAGATCTCGTCTCATAACCTGAG	AA deletion detection<br>SA deletion detection<br>*Hae*III site polymorphism<br>*Dpn*II site polymorphism
ry-10320-R	ACACACTTACCAATCAGCGGACG	BX deletion detection
ry-506-7640-F	CACCAGCTCGTGGAGCGCGTG	BP deletion detection
ry-PvuI-R	GGCTGACCCGATCGAAATACGCCTTG	BP deletion detection
ry-NdeI-F	GGAAAATCCATATGTAGGTTTCTTTATGCATACC	SA deletion detection
ry-9085-F	GGAATGGCCGTACTGGATGCGTG	*Hae*III site polymorphism<br>*Dpn*II site polymorphism
ry-EcoRI-F	GAGCGCAGCGAATTCCAGCCCTTG	*Pvu*II site polymorphism
ry-1460-R	CCGAGCGGCTCTTTGGGCC	*Pvu*II site polymorphism
ry-BstNI-F	CACCAAGCTGGATGCCAGTGAAG	*Bst*NI site polymorphism
ry-NdeI-R	GGTATGCATAAAGAAACCTACATATGGATTTTCC	*Bst*NI site polymorphism
ry-6000-F	GTGGGCTTCACCAAGGAGGGTC	*Kpn*I site polymorphism
ry-842-FLP-F	5HEX/CCTTTTAGATTAAGAAAAATTCAATGG	*Hae*III site polymorphism
ry-842-FLP-R	TTCATCGGAAAACTTGCTCA	*Hae*III site polymorphism
ry-1985-FLP-F	56-FAM/CCATAACGGCAGCACACTG	Polymorphism at +195
ry-1985-FLP-R	CTCAACGCCACCAATCAA	Polymorphism at +195

The last 4 entries were used in assays by high-resolution length analysis of the PCR products, and the forward primer (F) carried a fluorescent label in each case.
